# Granulomatous interstitial nephritis with CTLA-4 haploinsufficiency: a case report

**DOI:** 10.1186/s12882-022-02999-x

**Published:** 2022-11-16

**Authors:** Kaori Kohatsu, Tomo Suzuki, Madoka Takimoto, Katsuomi Matsui, Akinori Hashiguchi, Junki Koike, Sayuri Shirai

**Affiliations:** 1 Division of Nephrology and Hypertension, Department of Internal Medicine, St. Marianna University School of Medicine, Kawasaki, Kanagawa Japan; 2 Department of Nephrology, Kameda Medical Center, Chiba, Japan; 3 Department of Hematology, St. Marianna University School of Medicine Yokohama City Seibu Hospital, Yokohama, Kanagawa Japan; 4 Department of Nephrology and Hypertension, St. Marianna University School of Medicine Yokohama City Seibu Hospital, Yokohama, Kanagawa Japan; 5 Department of Pathology, Keio University School of Medicine, Tokyo, Japan; 6 Department of Diagnostic Pathology, St. Marianna University School of Medicine, Kawasaki, Kanagawa Japan

**Keywords:** CTLA-4 haploinsufficiency, Drug-induced interstitial nephritis, Granuloma, Acute kidney injury, Case report

## Abstract

**Background:**

Cytotoxic T lymphocyte antigen-4 (CTLA-4) is an essential inhibitory regulator of immune activation. CTLA-4 haploinsufficiency is known to be associated with dysregulation of FOXP3^+^ regulatory T cells, hyperactivation of effector T cells, and lymphocytic infiltration of multiple organs. However, there have only been a few reports of renal involvement with CTLA-4. Herein, we present a case of acute granulomatous tubulointerstitial nephritis (TIN) in a patient with CTLA-4 haploinsufficiency.

**Case presentation:**

A 44-year-old man presented with a 3-week history of fever and malaise, and subsequently developed acute kidney injury (AKI) a few days after treatment with levofloxacin (LVFX). A kidney biopsy and immunohistochemical staining revealed granulomatous TIN with dominantly infiltrating CD4^+^ T cells. General symptoms and renal impairment showed improvement after discontinuation of LVFX and initiation of oral steroids. However, they worsened following steroid tapering. Further, a colon biopsy analysis showed similar findings to the renal tissue analysis. We suspected that granulomatous TIN was possibly associated with CTLA-4 haploinsufficiency. Therefore, the patient was transferred to another hospital for further treatment of CTLA-4 haploinsufficiency using immunosuppressive agents.

**Conclusions:**

There have been few reports regarding renal involvement of CTLA-4 haploinsufficiency. In the present case, granulomatous TIN could have arisen due to instability of immune regulatory functions, such as CTLA-4 haploinsufficiency, and treatment with LVFX could have triggered immunologic activation and severe inflammation as well as renal dysfunction.

## Background

Cytotoxic T lymphocyte antigen-4 (CTLA-4) is an essential inhibitory regulator of immune activation, i.e., a “checkpoint” molecule in immune tolerance [1, 2]. Homozygous deficiency of *CTLA4* in mice causes fatal multiorgan lymphocytic infiltration and destruction [3–5]; however, the consequences of *CTLA4* mutations in humans are unknown. Human CTLA-4 haploinsufficiency is an autosomal dominant immune dysregulation syndrome with a heterozygous germline mutation in *CTLA4* that causes dysregulation of FOXP3^+^ regulatory T cells (Tregs), hyperactivation of effector T cells, and lymphocytic infiltration of multiple organs [6, 7]. Although several clinical symptoms such as autoimmune hemolytic anemia, immune thrombocytopenia, and lymphocytic infiltration in multiple organs have been reported in patients with CTLA-4 haploinsufficiency [6, 7], renal involvement has been rarely reported. Here, we report a case of granulomatous tubulointerstitial nephritis (TIN) with CTLA-4 haploinsufficiency.

## Case presentation

**Fig. 1:**
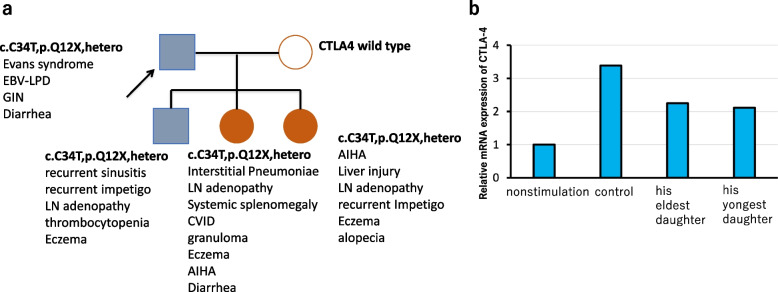
*CTLA4* mutation in our patient’s family and decreased *CTLA4* mRNA expression in his two daughters. **a** The family tree shows the same genetic mutation, heterozygous nonsense p. Q12X mutation in *CTLA4*, found in our patient (blue arrow) and his three children. His wife has wild type with no mutation. Their main clinical symptoms are described. **b** Real-time PCR using peripheral blood mononuclear cells (PBMCs), after stimulation with anti-CD3 and anti-CD28 obtained from his two daughters, showed decreased expression of the reactive *CTLA4* mRNA compared to that in the control group

A 44-year-old Japanese man presented with fever and malaise to the Department of Hematology at our hospital. He was prescribed medication for the common cold and levofloxacin (LVFX), 500 mg once daily, for a week; however, his fever did not subside. He presented to our hospital again and was prescribed the same medications for an additional week. However, the fever persisted, and the patient experienced loss of appetite. He was then admitted to our hospital for further examination and treatment. The patient had a prior history of immune thrombocytopenia at 19 years of age and of autoimmune hemolytic anemia at 32 years of age. During his late thirties, three of his children showed similar symptoms (Fig. 1a) and genetic testing revealed the same genetic mutation, a heterozygous nonsense p. Q12X mutation in *CTLA4*, in them. The expression level of reactive *CTLA4* mRNA in his two daughters, measured by real-time PCR, was decreased compared to that in the control group (Fig. 1b). Although a PCR test was not performed for the patient, he and his two daughters were diagnosed with CTLA-4 haploinsufficiency due to the presence of the same mutation.

On physical examination at admission, his temperature was 37.8 °C. The patient’s cardiovascular, pulmonary, abdominal, and neurological examinations were normal, and no edema, arthralgia, swelling, or pain in the cervical lymph nodes were reported.

His laboratory data on admission are described in Table 1. His serum creatinine level was significantly elevated from the baseline level of 0.8 mg/ dL. No occult blood, proteinuria, pyuria, or urinary casts were detected on urinalysis. However, tubular injury markers were elevated. After admission, oral LVFX was discontinued, and piperacillin-tazobactam was initiated to treat the persistent fever. However, the fever did not subside, and serum creatinine levels increased to 4.23 mg/dL on the fourth day of admission. He was then referred to our department.

**Table 1:** Laboratory findings on admission

CBC	
WBC (/uL)	10,800
RBC (/uL)	3.75 × 10^6^
Hb (g/dL)	9.9
MCV (fL)	81.9
PLT (/uL)	29.7 × 10^4^
Biochemistry	
TP (g/dL)	6.1
Alb (g/dL)	3.4
AST (IU/L)	21
ALT (IU/L)	16
LDH (IU/L)	423
BUN (mg/dL)	21.2
Cr (mg/dL)	3.18
eGFR (ml/min/1.73m^2^)	18.5
CRP (mg/dL)	10.75
Immunology	
IgA (mg/dL)	139
IgG (mg/dL)	649
IgM (mg/dL)	44
C3 (mg/dL)	92
C4 (mg/dL)	27
RF (IU/ml)	< 3
ANA	< 40
MPO-ANCA(U/ml)	< 1.0
PR3-ANCA (U/ml)	< 3.5
ASO (IU/ml)	51
Infection	
Blood culture	(−)
Urine culture	(−)
Urinalysis	
occult blood	(−)
Protein	(+)
Glucose	(−)
WBC	1–4/HPF
RBC	< 1/HPF
granular cast	(+)
waxy cast	(+)
protein quantity (g/gCr)	0.26
NAG (U/L)	12
α1-MG (mg/dL)	66.2
β_2_-MG (mg/dL)	37,720

The laboratory data on admission are shown. Inflammatory markers were elevated. IgG did not change remarkably compared to baseline levels. Upon urinalysis, occult blood, pyuria, or any casts were not detected; however, mild proteinuria and elevated tubular injury markers were found.

**Fig. 2:**
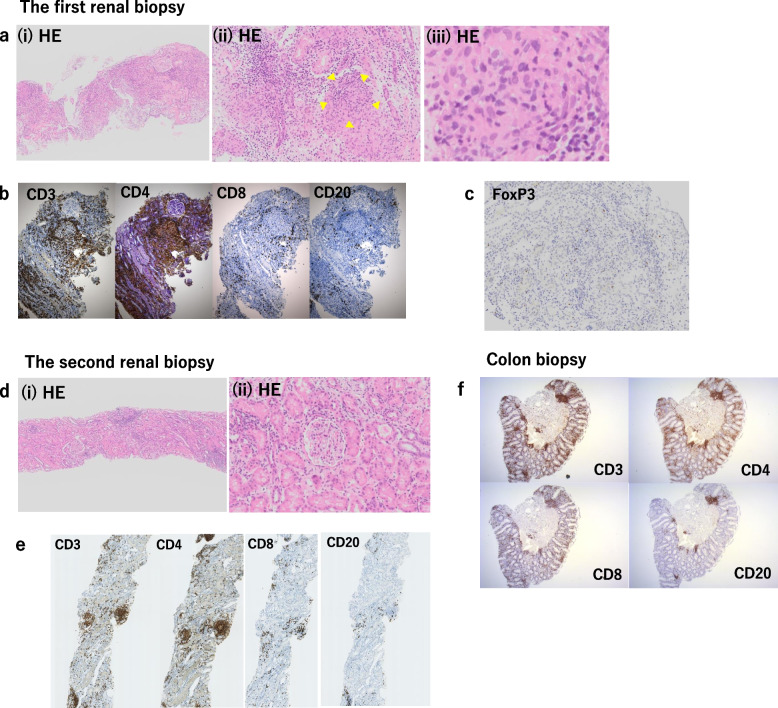
Pathological findings of renal and colon biopsy. (**a**-**i**) Light microscopy of the first renal biopsy at low power showing diffuse interstitial infiltrating of lymphocytes. (a-ii) Numerous lymphocytes with foreign body giant cells associated with non-caseating granulomas and a few eosinophils and plasma cells (yellow arrowheads). (a-iii) Granuloma formation at high power field. **b** Immunohistochemistry showing a more dominant infiltration of CD3^+^ and CD4^+^ T cells in the interstitial area compared to CD8^+^ and CD20^+^ T-cells. c FOXP3 staining of the first renal biopsy, wherein the proportion of FOXP3^+^ cells within CD4^+^ T cells was not as high. **d** Improved interstitial inflammation and diminished granuloma in the second renal biopsy compared to that in the first biopsy using light microscopy at low power (d-i), and high power (d-ii). **e** Dominant CD3^+^ and CD4^+^ T cell infiltration in the interstitial area in both the first and second renal biopsy, even though the stained area was decreased in the second biopsy compared with that in the first biopsy. **f** Colon biopsy showing CD4^+^ cells as the dominant lymphocytes infiltrating the intestinal mucosa in immunohistological staining, similar to the findings in the kidney

A kidney biopsy was performed on the seventh day. The specimen contained 12 glomeruli, of which one was globally sclerosed Light microscopy showed diffuse interstitial inflammation composed of numerous lymphocytes with foreign body giant cells associated with non-caseating granulomas, few eosinophils, and plasma cells. Tubulitis, with no evidence of vasculitis, was also observed. All other glomeruli, excluding the sclerosed, showed no significant changes (Fig. 2a). Immunofluorescence revealed non-specific findings, and electron microscopy revealed no electron-dense deposits. Ziehl–Neelsen acid-fast staining was negative. Immunohistological staining showed CD3^+^ and CD4^+^ T cells dominantly infiltrating the interstitial area (Fig. 2b). Moreover, immunostaining for FOXP3, the master transcription factor of Tregs, showed a decrease in the percentage of FOXP3^+^ cells within CD4^+^ T cells was decreased (Fig. 2c).

Based on the histopathological findings, the patient was diagnosed with granulomatous TIN. We thus speculated that granulomatous TIN was either associated with the use of LVFX or with the presence of CTLA-4 haploinsufficiency.

**Fig. 3:**
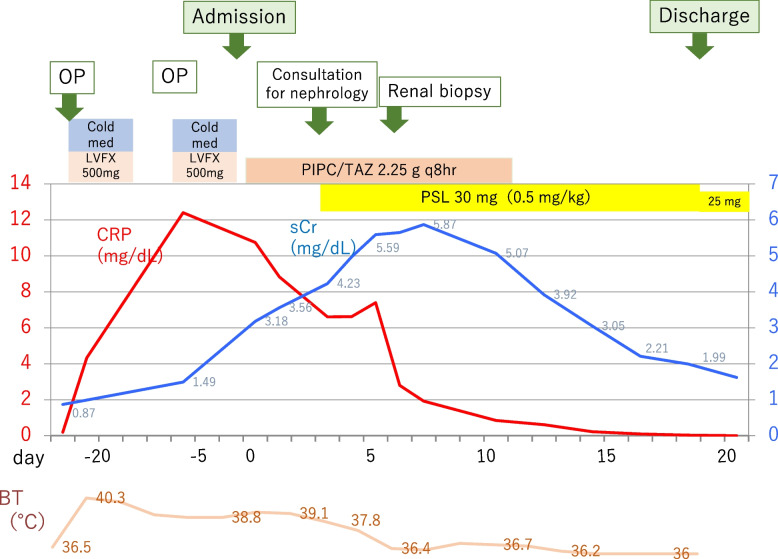
Clinical course during hospitalization. Although piperacillin-tazobactam was initiated after admission, the fever did not subside, and serum creatinine levels increased, then he was referred to our department. A kidney biopsy was performed on the 7th hospital day, and oral prednisolone (PSL) therapy, 30 mg (0.5 mg/kg) daily was initiated. After treatment, the fever subsided, and renal function improved gradually. The serum creatinine level decreased to 1.99 mg/dL within 2 weeks. Patient was discharged on the 17th day

Clinical course during hospitalization is shown in Fig. 3. We initiated oral prednisolone (PSL) therapy, 30 mg/day (0.5 mg/kg of body weight). After treatment, the fever subsided, and renal function improved gradually. The serum creatinine level decreased to 1.99 mg/dL within 2 weeks. The patient was discharged on the 17th day, and the dose of PSL was tapered to 25 mg/day. During outpatient follow-up, serum creatinine levels gradually improved, but did not decrease beyond 1.0–1.4 mg/dL even after PSL was tapered to 10 mg/day; moreover, malaise and arthralgia reoccurred. To re-evaluate the pathophysiology, we performed a second biopsy 5 months after the first. The results indicated that interstitial inflammation had improved and granuloma had diminished compared to that in the first biopsy (Fig. 2d). Similar to the first biopsy, CD3^+^ and CD4^+^ T cells predominantly infiltrated the interstitial area. However, in all immunohistological staining, the stained area was decreased (Fig. 2e) compared to that in the first biopsy. We evaluated five nonoverlapping interstitial fields in each tissue stained with CD3. CD3 positive areas were measured with the WinRoof Image Analyzer, version 4.3.0 (Mitani, Tokyo, Japan) and we compared the ratio of CD3 positive area relative to the entire area between the first and the second biopsy. As a result, there is significant decrease in the ratio of CD3 positive area in the second biopsy(9.6% ± 2.1) compared with the first(46.8% ± 5.3)(*p* = 0.043).

A colon biopsy was also performed as the patient reported diarrhea. The results showed lymphocytes infiltrating the intestinal mucosa; furthermore, CD4^+^ cells were the dominant lymphocytes (Fig. 2f). This was similar to the findings of the first renal biopsy.

The patient was then transferred to the same hospital as his children to receive further therapeutic intervention for CTLA-4 haploinsufficiency. In the other hospital, the patient was treated with abatacept, a recombinant fusion protein comprising the extracellular domain of human CTLA4, which inhibits the activation of T cells. PSL was maintained at 5 mg/day. After stating abatacept, his general symptoms (malaise, arthralgia) significantly improved and his creatinine level stayed around 1.0–1.2 mg/dl without more elevation.

## Discussion and conclusions

Here, we present a case of granulomatous TIN in a patient with CTLA-4 haploinsufficiency. It was difficult to clearly distinguish between drug-induced tubulointerstitial nephritis (DI-TIN) and renal involvement due to CTLA-4 haploinsufficiency.

The incidence of granulomatous TIN varied in different regions, ranging from 17.5 to 44.7%, and was most commonly caused due to exposure to drugs such as antibiotics [8–11]. In many case reports, granulomatous TIN has been attributed to the intake of penicillin and cephalosporins. Fluoroquinolones have been rarely reported to cause granulomatous TIN [12].

DI-TIN typically occurs within 3 weeks of initiating therapy with the causative drug in 80% of cases, with an average delay of 10 days following antibiotic use. It is characterized by mild proteinuria, sterile pyuria, and external manifestations that indicate systemic reactions such as fever, skin rash, and eosinophilia [13]. However, the detailed pathogenesis of DI-TIN, which represents the most common form of acute interstitial nephritis, remains unclear. There is a paucity of data regarding T-lymphocyte subsets that infiltrate the interstitial lesions. Therefore, a distinct diagnosis of DI-TIN seems difficult based on pathological characteristics alone. In our patient, LVFX had been used 2 weeks before the development of AKI. However, fever and elevated levels of inflammation markers were observed before the initiation of LVFX, and the general symptoms worsened following steroid tapering without any additional drug use, which suggested the involvement of other factors aside from LVFX use as the causation of granulomatous TIN.

Patients with CTLA-4 haploinsufficiency are reported to have lymphocytic infiltration of organs and lymphadenopathy. T cell infiltration in multiple organs such as the intestine, lung, bone marrow, and the central nervous system has also been reported [7]. However, renal involvement is very rare and its histological findings are limited [6].

Recently, immune checkpoint inhibitors, which are monoclonal antibodies directed against immune checkpoint proteins, have been increasingly used in the treatment of various cancers. Ipilimumab, an anti-CTLA-4 antibody has been approved for treating unresectable or metastatic melanoma and has shown favorable tumor response and extended patient survival [14]. Immune checkpoint inhibitors also have been known to induce side effects called “immune-related adverse events”. Several reports have described renal disorders related to ipilimumab, which mostly occur as AKI [15–20], but rarely as a nephrotic syndrome [21]. Renal biopsy showed acute interstitial nephritis with or without granuloma, and immunohistochemistry showed extensive T cell-dominant interstitial infiltrates that were mainly positive for CD3 and CD4 T cells [19, 20]. These results are similar to those in our patient. It could thus be considered that patients with *CTLA4* inhibition caused by ipilimumab show pathological and clinical features almost similar to those of patients suffering from CTLA-4 haploinsufficiency.

In previous reports of heterozygous germline mutations in *CTLA4* [6, 7], a high number of CD4^+^ and CD3^+^ cells infiltrated several organs, which was consistent with the findings in our patient. It was assumed that Tregs lost their suppressive capacity and that uncontrolled activation of auto-reactive T cells occurred due to CTLA-4 haploinsufficiency and contributed to inflammatory cell infiltration, in this case. These cells then migrated and infiltrated the target tissue via tissue self-antigen-specific T cell activation [22]. However, there are contradicting findings regarding the frequency of FOXP3^+^ Tregs within CD4^+^ T cells. Schubert et al. showed increased frequency compared to the control group [7]. On the contrary, Kuehn et al. reported significantly fewer FOXP3^+^ and CD25^+^ cells in peripheral blood of patients with CTLA-4 haploinsufficiency than in healthy donors, which were similar to our results, although their results were obtained using peripheral blood [6].

There are few reports of FOXP3 staining on kidney tissue in patients with acute interstitial nephritis as an adverse effect of CTLA-4 inhibitor. In a patient treated with a combination of nivolumab (anti-programmed cell death-1 antibody) and ipilimumab (anti-CTLA-4 antibody), immunohistochemistry showed extensive T cell-dominant infiltrates and FOXP3^+^ regulatory T cells in the interstitium; however, the frequency was not reported [19].

Therefore, it remains unclear if the degree of FOXP3-positive staining is associated with the function of FOXP3-positive regulatory T cells and the pathology or activity of CTLA-4 haploinsufficiency. It is also assumed that the percentage of FOXP3-positive cells in CD4^+^ T cells may vary during each inflammatory phase. Unfortunately, we could not quantify FOXP3 levels in renal biopsy or the frequency of Treg cell phenotypes in blood. This is a limitation in this case study and further studies are needed to understand the extent of Treg dysfunction.

In conclusion, we report a very rare case of granulomatous TIN with CTLA-4 haploinsufficiency. In this case, granulomatous TIN was suggested to be more likely associated with CTLA-4 haploinsufficiency, rather than with DI-TIN. Additionally, due to immune regulatory instability caused by CTLA-4 haploinsufficiency, treatment with LVFX could have triggered immunologic activation and severe inflammation as well as renal dysfunction.

## Data Availability

Not applicable.

## Abbreviations

AKI: acute kidney injury
CTLA-4: cytotoxic T lymphocyte antigen-4
DI-TIN: drug-induced tubulointerstitial nephritis
TIN: Tubulointerstitial nephritis
LVFX: levofloxacin
PBMC: peripheral blood mononuclear cells
PSL: prednisolone
Tregs: regulatory T cells
